# Long-term peritoneal dialysate exposure modulates expression of membrane complement regulators in human peritoneal mesothelial cells

**DOI:** 10.3389/fmed.2022.972592

**Published:** 2022-12-20

**Authors:** Kazuma Kobayashi, Toshikazu Ozeki, Hangsoo Kim, Masaki Imai, Hiroshi Kojima, Daiki Iguchi, Sosuke Fukui, Masafumi Suzuki, Yasuhiro Suzuki, Shoichi Maruyama, Yasuhiko Ito, Masashi Mizuno

**Affiliations:** ^1^Department of Nephrology, Nagoya University Graduate School of Medicine, Nagoya, Japan; ^2^Department of Renal Replacement Therapy, Nagoya University Graduate School of Medicine, Nagoya, Japan; ^3^Department of Immunology, Nagoya City University Graduate School of Medicine, Nagoya, Japan; ^4^Department of Nephrology and Rheumatology, Aichi Medical University, Nagakute, Japan

**Keywords:** peritoneal dialysis, complement, complement regulator, peritoneal dialysate, mesothelial cells

## Abstract

The membrane complement regulators (CRegs) CD46, CD55, and CD59 are highly expressed on human peritoneal mesothelial cells. However, how mesothelial CRegs change according to the peritoneal dialysis (PD) history of patients has remained unclear. We therefore examined longitudinal changes in CRegs in primary cultured mesothelial cells from PD patients (human peritoneal mesothelial cells; HPMCs) and examined which components of PD fluid (PDF) affect CRegs *in vitro*. We measured levels of soluble C5b-9 in overnight-dwelling PDF in PD patients and also evaluated changes in CRegs expression on HPMCs collected from PDF using flow cytometry and polymerase chain reaction at a 1-year interval of PD therapy. We also evaluated changes in CReg expressions with stimulation by each component of PDF (glucose, lactic acid and pH) using the Met5A human mesothelial cell line. Levels of sC5b-9 in PDF decreased significantly during 1 year, while expressions of CD46 and CD59 proteins and mRNAs increased significantly in HPMCs during 1 year. Analyzing Met-5A cells, we observed that expressions of the three CRegs were increased by glucose and lactic acid in a concentration-dependent manner, but conversely that expressions of CRegs were decreased by lower pH stimulation. History of PD might influence expression of CRegs by HPMCs through properties of PDF such as glucose, lactic acid, and pH. These results suggest that mesothelial cells may alter expression of CRegs for the purpose of protecting the peritoneum and the presence of PDF might affect peritoneal homeostasis associated with the complement system.

## Introduction

Peritoneal dialysis (PD) is one option for renal replacement therapy and allows patients to undergo home therapy. Compared with hemodialysis (HD), changes in body volume and uremic toxin levels are known to be less severe and host homeostasis is more maintained with PD. To maintain abdominal homeostasis in the host, the immune system plays important roles such as removing “foreign” materials from the peritoneal cavity, repairing injured peritoneum and so on (1). However, in PD patients, uremic conditions, exposure to PD fluid (PDF), catheter insertion associated with PD therapy and incidence of peritonitis may hinder maintenance of peritoneal homeostasis, including by the immune system, in PD patients (1). The complement (C) system is an important partner of the immune system in the host. As part of the innate immune system, the C system protects the host from invading microorganisms, scavenges foreign bodies, and also modifies the immune system to maintain homeostasis in the host (2). The C system is controlled by the balance of activation systems and C regulators to prevent unexpected activation of the C system, as inappropriate C activation can result in local and/or systemic inflammation and damage to the host (2–4). In the peritoneum, production of C proteins such as C3 and C4 has been reported and the C system is expected to play important roles in the peritoneum (5, 6). Concerning systemic circulation in PD patients, the membrane C regulator (CReg) CD59 might be modified on red blood cells under hyperglycemic conditions and C activation products can be deposited in the vascular endothelium in PD patients (7, 8). Of note, in addition to systemic expressions of CRegs, the mesothelial membrane CRegs CD46, CD55, and CD59 are broadly distributed in the peritoneum (9–13), and play important roles in the maintenance of peritoneal homeostasis in humans and animal models (11, 12). In addition, in mesothelial cells harvested from peritoneal lavages from PD patients, changes in CReg CD55 expression were associated with peritoneal function and decreased CD55 expression might diminish suppression of C activation in mesothelial cells (13, 14).

However, changes in CReg expressions with long-term PD therapy have not yet been clarified, because our previous study was cross-sectional in design. We therefore investigated changes in CReg expressions for peritoneal mesothelial cells in patients during 1 year of PD and examined which characteristics of PDF might affect CReg expressions.

## Materials and methods

### Antibodies and agents

Mouse monoclonal antibodies (mAbs), anti-human CD55 (clone 1C6) and anti-human CD59 (clone 1F5), were characterized as previously reported (15, 16). The mAb anti-human CD55 (clone 1C6) was kindly gifted by Dr. T. Fujita (Fukushima, Japan). To detect human CD46, mAb anti-human CD46 (clone J4-48) and mAb anti-human CD46 (clone MEM-258) were purchased from Abnova (Taipei, Taiwan) and Bio-Rad Laboratories (Hercules, CA, USA), respectively. As an isotype-matched control for fluorescence-activated cell sorting (FACS) analysis, purified mouse immunoglobulin (Ig)G1 (Life Technologies, Camarillo, CA, USA) was used. Fluorescein isothiocyanate (FITC)-labeled goat anti-mouse IgG was purchased from Jackson Immuno Research Laboratories (West Grove, PA, USA). When the lot of antibodies or antibodies against CD46 were changed, staining was performed for 14 selected samples using both the antibodies before and after the change, and differences were corrected using linear regression (*p* < 0.0001, *R*^2^ = 0.9253).

Both D-glucose and D-mannitol were purchased from Sigma-Aldrich (St. Louis, MO, USA). Sodium lactate, sodium bicarbonate, and hydrochloric acid were purchased from FUJIFILM Wako (Osaka, Japan).

### Collection of profiles, demographic data and PDF samples from end-stage renal disease (ESRD) patients on PD therapy

The present experiments were approved by the ethics committee at the Institute of Nagoya University Hospital. We used all samples from 44 patients who started PD between August 2006 and February 2019 in Nagoya University Hospital and provided written agreement to the hospital for the use of PDF samples and demographic/laboratory data in the present study. Dialysate-to-plasma creatinine concentration ratio (D/P Cr) was obtained by peritoneal equivalent test to evaluate peritoneal function (17).

Sampling of human peritoneal mesothelial cells (HPMCs) and the following experiments were performed twice at an interval of 1 year. For each time point/patient for analysis of CReg expressions in primary mesothelial cells cultured from PDF (human peritoneal mesothelial cells; HPMCs), we collected PDF twice during 1 month. The first harvested PDF sample was centrifuged and the supernatant was stored at −80°C until enzyme-linked immunosorbent assay (ELISA) analysis.

### Primary cultured HPMCs in ESRD patients on PD from dwelling lavages

Human peritoneal mesothelial cells were obtained by centrifugation of overnight-dwelling PDF taken randomly from clinically stable patients using previously described methods (14, 18–20). These PDF samples were harvested from PD patients who had not suffered infectious peritonitis for at least 3 months prior to harvesting PDF. Briefly, HPMCs were isolated using low-speed (200 × *g*) centrifugation, washed with RPMI-1640 (Sigma-Aldrich), then cultured in RPMI-1640 containing L-glutamine (Sigma-Aldrich) supplemented with 15% fatal bovine serum (Sigma-Aldrich), insulin/transferrin/selenium A (Invitrogen, Tokyo, Japan), 10^–5^ M of 2-mercaptoethanol (FUJIFILM Wako, Osaka, Japan), and 400 μg/L of hydrocortisone (Sigma-Aldrich) in humidified air with 5% CO_2_ at 37°C. Cells reached confluence in 7–10 days, and were split two to three times, with second passage cells used for the following experiments.

### FACS analysis of CReg expressions in HPMCs

To detect expressions of CD46, CD55, and CD59 in human mesothelial cells, primary culture cells were first incubated with the respective mAb or mouse IgG1 as an isotype-matched control, followed by incubation with FITC-anti-mouse IgG. After centrifugation at 200 × *g* for 5 min at room temperature, the pellet was adjusted to 0.4 × 10^6^cells/mL and incubated with anti-human CD46, CD55 or CD59 mAb, followed by incubation with FITC-labeled goat anti-mouse IgG (Jackson ImmunoResearch Laboratories, West Grove, PA, USA). To eliminate the effects of dead cells, cells were stained with 2.5 μL of 7-aminoactinomycin D (7-AAD) (BD Pharmingen, San Jose, CA) and incubated for 10 min on ice in the dark. The mean fluorescence intensity (MFI) of CRegs was evaluated under FACS analysis on a BD FACS-Canto II™ (Becton Dickinson, San Jose, CA). Experiments were repeated twice using independent HPMCs for each patient within 8 weeks. Values of MFI represent the mean MFI of three independent experiments for each patient according to our previous report (14). For reproducibility, we calibrated the cytometer using Spherotech 8 Peak Validation Beads (BD Pharmingen, BD Biosciences). Because the mAb anti-CD46 (clone J4-48) was discontinued, the mAb anti-human CD46 (clone MEM-258) was used instead. As shown in Supplementary Figure 1, the MFIs between clones J4-48 and MEM-258 are well correlated, so we used the adjusted MIF between both clones in the present study.

### Polymerase chain reaction (PCR) and expression of mRNA of CRegs in human mesothelial cells

RNA was prepared from cultured cells using an RNeasy Mini Kit (Qiagen Japan, Tokyo, Japan), and first-strand cDNA was synthesized using a High Capacity Reverse Transcription Kit (Applied Biosystems, Foster City, CA), according to the instructions from the manufacturer. Total RNA (1 μg) was then reverse-transcribed. To validate gene expression changes, quantitative real-time reverse transcription PCR (RT-PCR) analysis was performed with a Prism 7500HT sequence detection system (Applied Biosystems) using Taq-Man gene expression assays for human CD46 (Hs00611257_m1), CD55 (Hs00167090_m1), CD59 (Hs00174141_m1), and 18S ribosomal RNA (4319413E) according to the specifications of the manufacturer (Applied Biosystems). Thermal cycler conditions were as follows: hold for 10 min at 95°C, followed by two-step PCR for 40 cycles of 95°C for 15 s and 60°C for 1 min. All reactions were performed in duplicate. Amplification data were analyzed using Sequence Detection Software version 1.4.1 (Applied Biosystems), with 18S ribosomal RNA used as an endogenous control.

### ELISA for measurements of human soluble C5b-9 (sC5b-9) in PDF

To investigate whether levels of the C activation product sC5b-9 in PDF could be influenced by duration of PD therapy, we measured levels of sC5b-9 from overnight-dwelling PDF from 44 patients on PD therapy.

To measure levels of human sC5b-9 in PDF, a MicroVue™ sC5b-9 plus EIA kit (Quidel, San Diego, CA, USA) was used. Total protein amounts in PDF were also measured using a BCA protein assay reagent (Thermo Fisher Scientific, Waltham, MA, USA). ELISA assays and measurements of protein amounts were performed according to the instructions provided by manufacturers. All samples were measured in duplicate and mean values were used. Each value was adjusted for total protein level in PDF according to the following formula: adjusted level of sC5b-9 (ng/μg) in PDF = {[level of sC5b-9 (ng/mL) in PDF]/[protein level in PDF (μg/mL)]} × 1000.

### *In vitro* experiments using a human mesothelial cell line

The Met-5A human mesothelial cell line, derived from pRSV-T-transfection of cells from pleural fluid of non-cancerous patients, was purchased from the American Type Culture Collection (ATCC, Manassas, VA, USA) and maintained according to ATCC guidelines. After becoming semi-confluent, MeT-5A cells were stimulated by changing the medium. D-glucose or D-mannitol as osmotic gradients used in PDF, and sodium lactate or sodium bicarbonate as buffers used in PDF were added to the culture medium, and expressions of CRegs on the cell surface after 24 or 72 h were compared by FACS. Concentrations of glucose, lactic acid and bicarbonate were determined with reference to the available PDF. To investigate changes in CReg expressions with buffer in PDF, Met-5A cells were stimulated by medium supplemented with concentrations of lactate and/or bicarbonate with reference to Dianeal-N^®^ or Reguneal^®^. Met-5A cells were also stimulated with pH 5.0 culture medium adjusted with hydrochloric acid, equivalent to the pH of conventional acidic PDF. After 24 h, CReg expressions were analyzed and compared to medium only (control). Since cell death occurred with long-term acidic stimulation, the stimulation time was set to 24 h. Each *in vitro* experiment was independently repeated 3 times. In a set of experiments, each sample was measured in triplicate and the mean was used for each value.

### Statistical analysis

All values are expressed as mean ± standard deviation (SD). Comparisons over 1 year were performed using the Wilcoxon matched-pairs signed rank test. For *in vitro* studies, comparisons among multiple groups were performed using the Kruskal–Wallis test and Dunn’s *post-hoc* test. Comparisons between groups were performed using the Mann–Whitney test. Values of *P* < 0.05 (5%) were considered statistically significant. GraphPad Prism version 8.1.2 software (GraphPad Software, San Diego, CA, USA) was used for all statistical analyses.

## Results

### Baseline characteristics and changes in laboratory data including D/P Cr with 1 year of PD therapy

**FIGURE 1:**
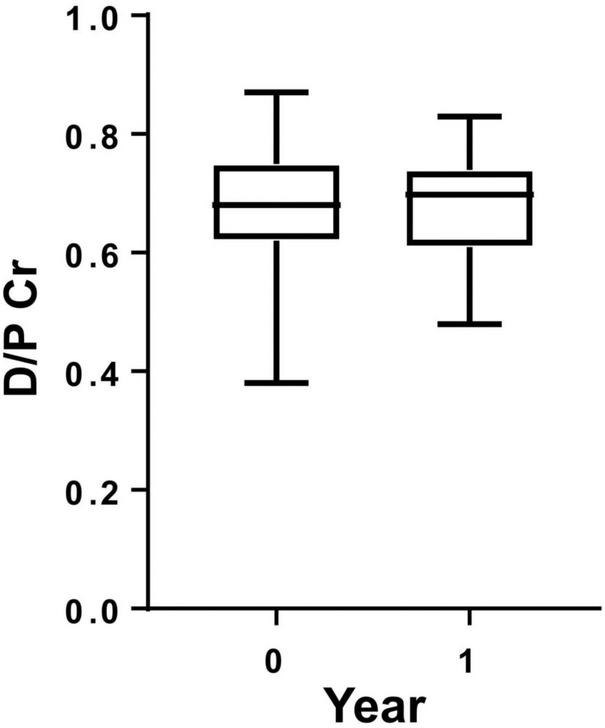
Dialysate-to-plasma creatinine ratio (D/P Cr) in patients on peritoneal dialysis. D/P Cr did not show any significant change during 1 year of observation.

The medical backgrounds of 44 PD patients are shown in Table 1. D/P Cr measured at intervals of 1 year did not show any change (Figure 1). Tiny but significant improvements were seen after 1 year for serum albumin (Alb) and blood hemoglobulin (Hb) levels, and significant differences were also seen for weekly renal Kt/V (urea clearance) as a proxy for residual renal function and weekly peritoneal Kt/V urea as a proxy for required PD. These results support the notion that decreased residual renal function was observed over the course of 1 year and, instead of decreased residual renal function, an increase in total PD was required.

**TABLE 1:** Background characteristics of peritoneal dialysis (PD) patients (*n* = 44).

Characteristic		Baseline (*n* = 44)	One-year follow-up	*P*-value
Peritoneal dialysis history (months), mean (SD)		35.9 (38.0)		
Age (years old), mean (SD)		62.5 (12.4)		
Body mass index (kg/m^2^), mean (SD)		22.6 (3.0)		
Gender, no. (%)	Male	26 (59.0)		
	Female	18 (41.0)		
Usage of icodextrin, no. (%)		14 (31.8)		
Background disease, no. (%)	Diabetes	11 (25.0)		
	Glomerulonephritis	18 (41.0)		
	Nephrosclerosis	6 (13.6)		
	Others	9 (20.5)		
Comorbidities, no. (%)	Diabetes	11 (25.0)		
	Hypertension	41 (93.1)		
Serum albumin level (g/dL), mean (SD)		3.2 (0.4)	3.3 (0.4)	0.035
Blood hemoglobin level (g/dL), mean (SD)		11.5 (0.9)	11.6 (0.8)	<0.0001
Serum β2-microglobulin (mg/L), mean (SD)		21.2 (7.1)	25.4 (11.6)	0.77
Dialysate-to-plasma creatinine ratio, mean (SD)		0.68 (0.11)	0.67 (0.10)	0.56
Weekly peritoneal Kt/V urea, mean (SD)		1.09 (0.38)	1.19 (0.41)	0.039
Weekly renal Kt/V urea, mean (SD)		0.69 (0.46)	0.63 (0.49)	0.0043

SD, standard deviation.

### Expressions of CD46 and CD59 on the surface of HPMCs increased with 1 year of PD therapy

**FIGURE 2:**
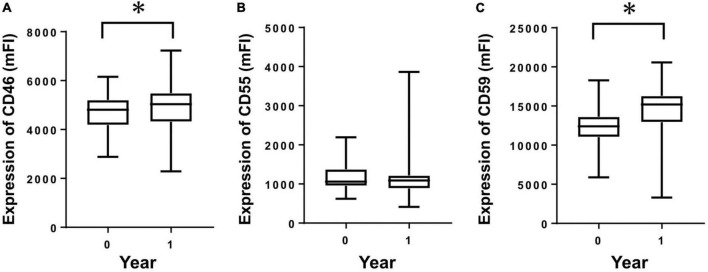
Expression of membrane complement regulators (CRegs) on human peritoneal mesothelial cells of PD patients during 1 year. Expressions of CRegs CD46 **(A)** and CD59 **(C)** were significantly increased during 1 year of observation. Expression of CReg CD55 **(B)** was not significantly changed. *P < 0.05.

To investigate longitudinal changes in CRegs on HPMCs of PD patients, we compared expressions of CRegs on HPMCs at two points within 1 year under FACS analysis. Expressions of CD46 and CD59 were significantly increased within 1 year (Figures 2A,C). CD55 did not show any significant change in 1 year (Figure 2B), although expression of CD55 tended to increase.

### Expressions of CD46 and CD59 mRNAs increased significantly with 1 year of PD therapy

**FIGURE 3:**
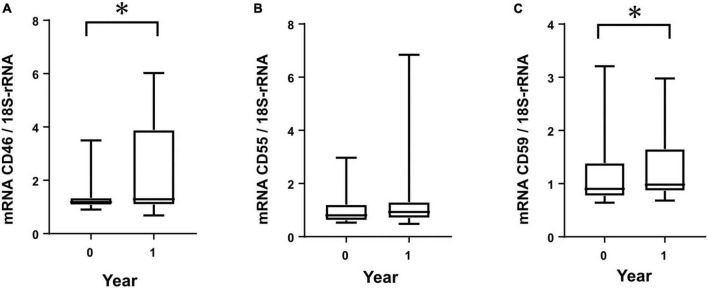
Membrane complement regulator (CReg) mRNA from human peritoneal mesothelial cells of PD patients during 1 year. Clear increases were seen for mRNA of CD46 **(A)** and CD59 **(C)**, but not CD55 **(B)**, during 1 year. *P < 0.05.

From the cultured HPMCs of PD patients, mRNA was extracted and analyzed from two points within a 1-year period. CD46 and CD59 mRNAs showed significant increases within 1 year (Figures 3A,C). CD55 did not show any significant change within 1 year, although a tendency toward increased mRNA levels was observed (Figure 3B).

### Levels of sC5b-9 in PDF decreased within 1 year of PD therapy

**FIGURE 4:**
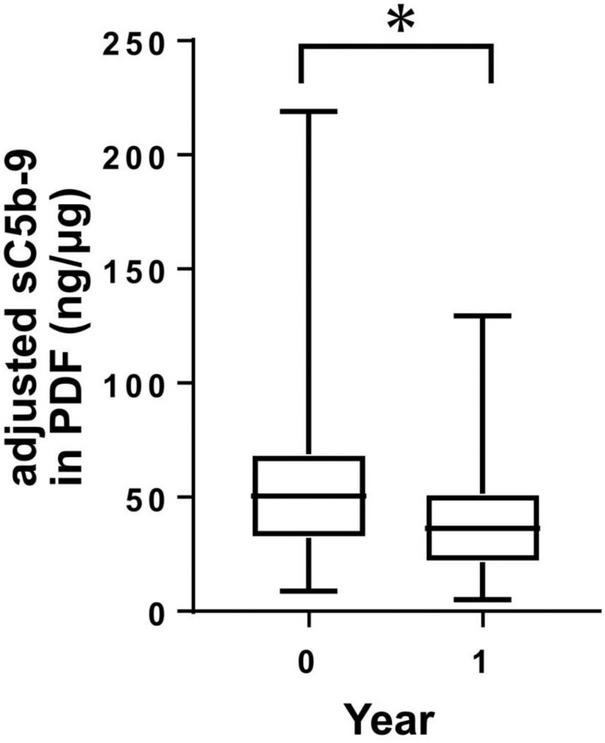
Levels of sC5b-9 in peritoneal dialysis (PD) fluid (PDF) from PD patients during 1 year. Levels of sC5b-9 in PDF significantly decreased during 1 year. *P < 0.05.

To investigate peritoneal changes in activation of the complement system in terms of changes in CReg expressions, we measured levels of sC5b-9 in PDF from PD patients, adjusted by protein levels (adjusted sC5b-9), at two points within the 1-year period by ELISA. Levels of adjusted sC5b-9 in PDF of PD patients were significantly decreased over the 1-year period (Figure 4).

### CRegs expressed on Met-5A mesothelial cells were increased by glucose and lactate stimulation, but decreased by acid stimulation

**FIGURE 5:**
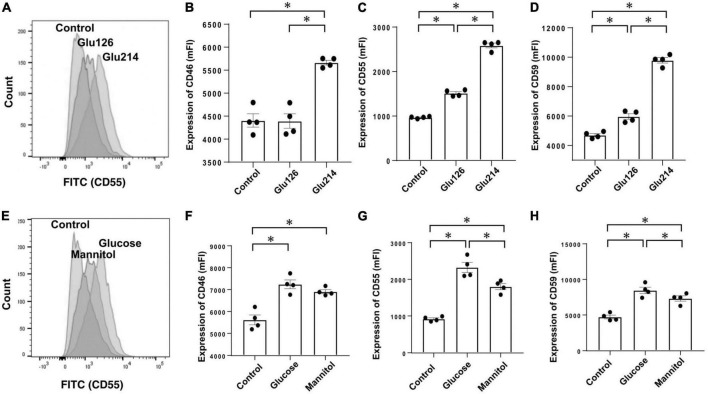
Expressions of membrane complement regulators (CRegs) of Met-5A cells stimulated with glucose and evaluation of osmotic effects. Expressions of CRegs in Met-5A cells increased dependent on glucose concentration **(A–D)**. When the effects of osmolar stimulation were evaluated between 214 mOsm/L of glucose (equivalent glucose concentration to 2.5% glucose-based peritoneal dialysate) and the identical osmolality of mannitol, CD55 and CD59, but not CD46, were significantly increased with glucose, as compared to with mannitol. **(A,E)** Show sample histograms for CD55 expression stimulated with different concentrations of glucose-supplemented medium or control and stimulated with the same osmolality of glucose or mannitol, respectively. **(B–D,F–H)** Show changes in expressions of CD46, CD55, and CD59, respectively. Graph sets of panel **(B–D,F–H)** show results of stimulation with different concentrations of glucose and results of stimulation by glucose or mannitol at the same osmolality, respectively. *P < 0.05.

We performed *in vitro* studies to investigate which components of PDF influence expressions of CRegs on mesothelial cells as *in vitro* studies using the Met-5A mesothelial cell line. First, we investigated the effects of glucose in Met-5A cells incubated with 126 mOsm/L, and 214 mOsm/L of glucose in culture medium, equivalent to glucose concentrations of 2.5% and 4.25% in glucose-based PDF. After 72 h, expressions of CRegs CD46, CD55, and CD59 increased with increasing glucose concentration (Figures 5A–D). However, expressions of CRegs were not changed with 24 h of incubation in glucose-added medium (either 126 or 214 mOsm/L) (data not shown). To analyze whether up-regulation of CRegs was caused by the glucose itself or the increased osmolarity in mesothelial cells, Met-5A cells were incubated with 214 mOsm/L of glucose or 214 mOsm/L of mannitol, both offering equivalent osmolarity to 4.25% glucose-based PDF. Expressions of CD55 and CD59 were significantly increased in both glucose- and mannitol-containing media compared to the control (Figures 5E–H), but were significantly higher in glucose-containing medium compared to mannitol. Expression of CD46 did not differ significantly between stimulation by 214 mOsm/L of glucose or 214 mOsm/L of mannitol, although expression of CD46 tended to be lower with mannitol stimulation than with glucose stimulation (Figure 5F).

**FIGURE 6:**
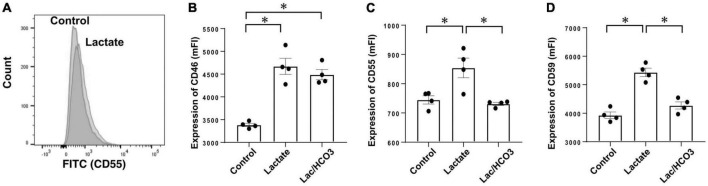
Expression of membrane complement regulators (CRegs) of Met-5A stimulated with lactate. **(A)** Shows a sample of histograms for expression of CD55 stimulated by sodium lactate-added culture medium and control medium. **(B–D)** Show that stimulation with lactate increased expressions of CRegs on the cell surface of MeT-5A cells, while co-stimulation with lactate and bicarbonate did not show any significant change. In the group stimulated with sodium lactate, the concentration of lactate was set at 40 mmol/L, while in the group stimulated with both bicarbonate and sodium lactate, the respective concentrations were set at 25 and 15 mmol/L in culture medium. Those concentrations were determined with reference to Dianeal-N dialysate solution and Reguneal dialysate solution. *P < 0.05.

Next, when we compared effects between 40 mEq/L of lactate buffer-based PDF (equivalent to Dianeal-N peritoneal dialysate) and 30 mEq/L of bicarbonate mixed with 5 mEq/L of lactate based-PDF (equivalent to Reguneal peritoneal dialysate) for expressions of CRegs in Met-5A cells, significant increases in expressions of CRegs CD46, CD55, and CD59 were observed with lactate stimulation (Figure 6). Compared with simple lactate stimulation in culture medium, expressions of CD55 and CD59 were significantly lower in lactate mixed with bicarbonate-based medium (Figures 6C,D), but expression of CD46 in lactate mixed with bicarbonate-based medium tended to be slightly decreased, although no significant change was seen compared with lactate-based medium (Figure 6B).

**FIGURE 7:**
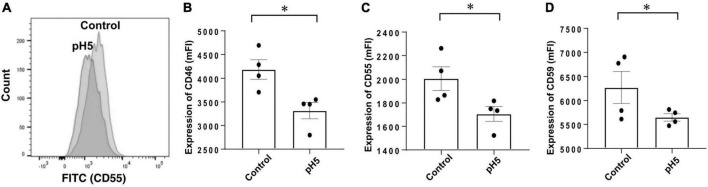
Expression of membrane complement regulators (CRegs) of Met-5A cells stimulated with acidic condition medium. **(A)** Shows a sample of histograms of expression for CD55 stimulated with pH 5.0-adjusted culture medium and control neutral medium (pH 7.4). **(B–D)** Show that stimulation with pH 5.0 acid medium decreased expression of CRegs on the cell surface of Met-5A. *P < 0.05.

Third, because acidic dialysate solution was sometimes used, the pH of the neutral medium was lowered for stimulation, and expressions of CRegs were analyzed. After acidic stimulation, CRegs on Met-5A were significantly decreased (Figure 7).

## Discussion

In PD patients, we observed that expressions of CD46 and CD59 were significantly increased on HPMCs within 1 year. At the same time, expression of CD55 was not significantly changed, although an increasing tendency was observed. In contrast, as a C activation product, levels of sC5b-9 in PDF were decreased after 1 year of observation, suggesting that C activation was more regulated according to increased CReg expressions and might protect against excessive autologous C activation in the peritoneal cavity of PD patients. We then focused on PDF and investigated whether individual features of PDF could influence expression of CRegs. When we investigated the effect of glucose as an osmotic agent in PDF using *in vitro* studies, up-regulation of CRegs in mesothelial cells was seen to depend on glucose levels in culture media. Greater CReg expressions were observed with glucose stimulation compared to equivalent osmotic mannitol stimulation. Therefore, glucose appears to directly induce increases in some CRegs, although glucose-induced increases in CRegs might be induced by the osmolarity of the medium being used. In addition, when we compared effects of exposure to sodium lactate only or a mixture of sodium bicarbonate and sodium lactate as equivalents of Dianeal-N PDF, a neutral solution, and Reguneal PDF, a newer, pH-adjusted PDF, we observed that up-regulation of CRegs in mesothelial cells was induced by sodium lactate only compared with the mixture of sodium bicarbonate with sodium lactate. In contrast, low pH-adjusted medium decreased expressions of CRegs in mesothelial cells exposed to low pH levels similar to conventional acidic PDF, supporting our previous report (21).

Our present data showed that, in a longitudinal analysis, up-regulation of CReg expressions by mesothelial cells was observed with 1 year of PD therapy in most PD patients with neutral PD solution, although changes over time to CReg expressions remained unknown for patients in our past cross-sectional study of 31 patients (14). Because we previously observed decreased CReg expressions in the peritoneal mesothelium of patients with PD therapy compared with mesothelial cells in peritoneum of patients with non-chronic kidney diseases in a cross-sectional study (14), changes in some CReg expressions might have occurred under continuous performance of PD therapy for 1 year in the present study. As a hypothesis, changes in CReg expressions might occur under uremic conditions and recovery of CReg expressions might be induced with improvement in a uremic condition after starting PD. In addition, the present increase in CReg expressions might have developed under long-term exposure to each element in PDF, such as glucose and lactate. From a past report regarding the up-regulation of CD59 expression in blood cells (7), exposure to glucose might influence increases in CD59 expression in mesothelial cells. Concerning lactate exposure, activation of the C system might be induced as an inflammatory process and progress to up-regulation of CRegs to protect injured peritoneum from autologous C attacks accompanying other inflammatory reactions (22).

With the use of conventional low-pH PDF, impairment of CD55 expression was shown in injured peritoneum (13). In peritoneal vessels under long-term PD therapy, impairment of CD59 was also reported and soluble CD59 was increased in PDF (23). Although CD55 expression correlated inversely with D/P Cr in our previous report (14), D/P Cr did not significantly change in the present cases during 1 year of observation, and no significant changes in CD55 expression were observed in HPMCs. In contrast, similar to our previous report (21), exposure to low-pH medium decreased expressions of CRegs. During the use of PDF, both increases and decreases in CRegs could be induced according to our results. As mentioned above, some discrepancies were observed in changes in CReg expressions between the present and previous reports. Expressions of CRegs may have differed with observation of patients for 1 year (Supplementary Figure 2). Another possibility is that components and factors other than FDP-related factors may be involved, such as silicone rubber catheters and uremic toxins. As another reason, unlike other reports, the present study used a neutral-pH PD solution, which may have influenced discrepancies in CReg expressions.

In the present study, we showed changes in CReg expressions in mesothelial cells. However, the reason for the changes in CRegs remains unknown. Until now, C3 and C4, and probably C5, C7, C8 and C9, have been reported as components of the C system in the peritoneum (9, 24). As a generator of C3 convertases in the alternative pathway, factor D is reported to be abundantly involved in the peritoneum according to a proteomic analysis (25). Previous *in vivo* experiments about CRegs (6, 10–12) identified peritonitis as a factor related to PD therapy that would likely influence CReg expressions and impairment of C system regulation in the peritoneal cavity might interfere with the homeostasis of host immunity. Changes in CReg expressions might contribute to the maintenance of peritoneal homeostasis associated with the C system. Under longitudinal observation in the present study with neutral PD solution, no significant changes in D/P Cr associated with peritoneal function were observed (26, 27), although acidic PD solutions are known to cause severe peritoneal injuries under long-term PD therapy, representing an important risk factor for the development of encapsulating peritoneal sclerosis as a lethal complication of PD therapy (28, 29). However, a recent Japanese cohort study using neutral PDF (26) and pathological studies (28, 29) have shown that peritoneal impairments such as mesothelial damage, vasculopathies and fibrosis were less pronounced in patients with neutral PDF compared with acidic PDF after long-term PD therapy. Because peritoneal changes were milder under neutral-pH PDF compared with conventional acidic PDF, expressions of CRegs might be less affected by neutral-pH PDF than by more acidic PDF. This may explain some of the discrepancies regarding changes in CRegs in past reports. Whether PD therapy might up- or down-regulate expressions of CRegs in mesothelial cells of the peritoneum has remained unknown. Probably, changes in CReg expressions might work in both directions, depending on conditions of the peritoneal cavity such as peritonitis, post-peritonitis, and short- or long-term PD history.

As we have previously shown, expression of CD55 in mesothelial cells of the peritoneum is decreased in ESRD patients compared with non-CKD patients on PD (14). Our speculation was that dialysis, such as removal of uremic toxins and adjustment of pH in the host, might recover the expressions of CRegs after starting PD with pH-adjusted PDF. In fact, levels of C3a, C5a, and sC5b-9 as the C activation product might be temporarily increased in the short term after starting PD, then subsequently decrease (30). This might be partly associated with increases in CRegs. As limitations, sample numbers were small and the observation period was still only 1 year. As shown in Supplementary Figure 2, when we observed changes in CReg expressions in each patient, some cases showed different behaviors for CReg expressions. Whether peritoneal sC5b-9 levels as a product of C activation were higher in ESRD patients than in healthy people also remains unclear, because we could not harvest peritoneal fluids. As another limitation, changes in CReg expressions remain unclear for longer-term PD therapy such as 10 years or more, due to insufficient data. Given those limitations, we will continue to follow PD patients and accumulate more data.

In conclusion, expressions of CRegs in HPMCs were affected by PD therapy in a time-dependent manner. We also showed that PDF might increase and decrease expressions of CRegs in mesothelial cells, suggesting that long-term exposure to PDF-related factors might not only induce up-regulation of some CRegs, but also possibly decrease expressions of other CRegs. Those changes in CReg expressions might contribute to adjustments in the balance of the C activation system in the peritoneum to improve C-system maintenance of peritoneal immunity. Further analyses are required to clarify influences on homeostasis in the peritoneal C system of ESRD patients under long-term PD.

## Data availability statement

The raw data supporting the conclusions of this article will be made available by the authors, without undue reservation, but it will be essential to get agreement of the Ethics Committee at the Institute of Nagoya University Hospital.

## Ethics statement

The present experiments were approved by the Ethics Committee at the Institute of Nagoya University Hospital. The patients/participants provided their written informed consent to participate in this study.

## Author contributions

KK, TO, HKo, YI, DI, and MM conceived and designed the research. KK, TO, MS, and DI performed the experiments. KK, TO, HKi, and MM analyzed the data. TO, YI, MM, HKi, HKo, YS, MI, SF, and SM interpreted the results of the experiments. TO and MM prepared the figures. YI, MM, HKi, and YS drafted the manuscript. TO, HKi, and MM edited and revised the manuscript. MM approved the final version of manuscript. All authors contributed to the article and approved the submitted version.

## Supplementary material

Supplementary Figure 1 Correlation between fluorescence intensity between mAbs MEM-258 and J4-48 as anti-human CD46. Monoclonal Ab MEM-258 correlated well with J4-48 to evaluate mean fluorescence intensity as CD46 expression in mesothelial cells. We therefore adjusted both according to the formula in the graph.

Supplementary Figure 2 Changes in membrane complement regulars (CRegs) CD46, CD55, and CD59 during 1 year of observation. **(A–C)** Show changes in expression of CD46, CD55, and CD59, respectively, for individual patients in 1 year.
