# Supplementary figures and images for: Long-term peritoneal dialysate exposure modulates expression of membrane complement regulators in human peritoneal mesothelial cells
Source: Front Med (Lausanne). 2022 Dec 20;9:972592. doi: 10.3389/fmed.2022.972592 (PMC9815709; doi:10.3389/fmed.2022.972592)

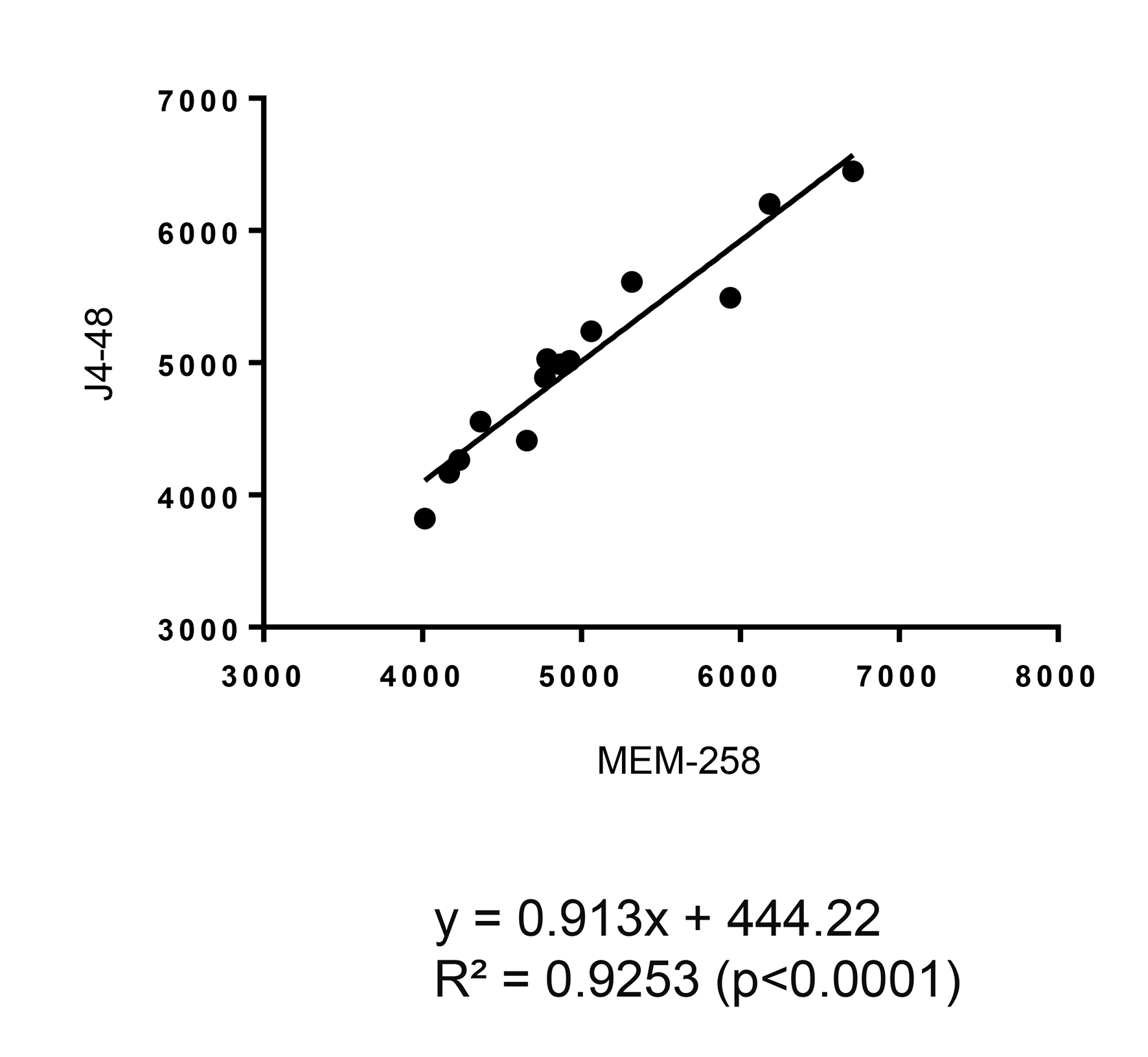

Supplement: Supplementary Figure 1 — Correlation between fluorescence intensity between mAbs MEM-258 and J4-48 as anti-human CD46. Monoclonal Ab MEM-258 correlated well with J4-48 to evaluate mean fluorescence intensity as CD46 expression in mesothelial cells. We therefore adjusted both according to the formula in the graph. [file Image_1.TIF]

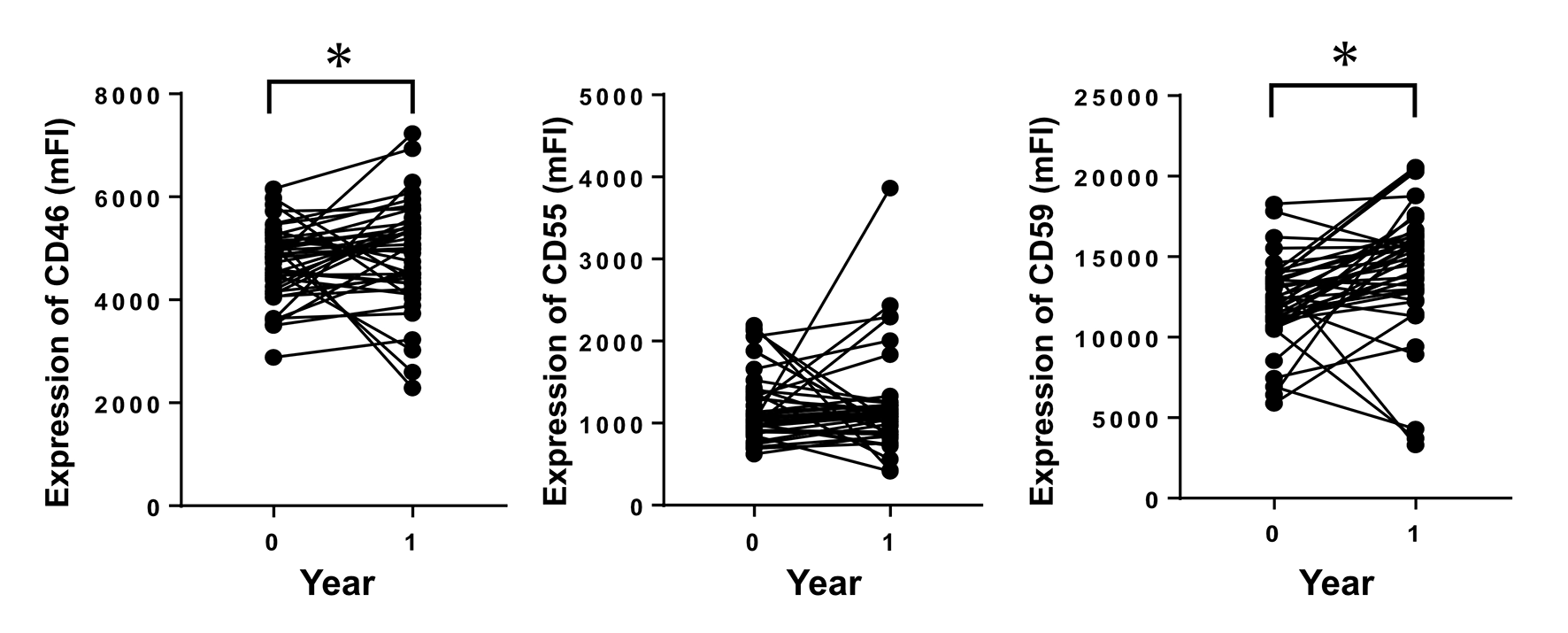

Supplement: Supplementary Figure 2 — Changes in membrane complement regulars (CRegs) CD46, CD55, and CD59 during 1 year of observation. (A–C) Show changes in expression of CD46, CD55, and CD59, respectively, for individual patients in 1 year. [file Image_2.TIF]
